# The clinical implications of using a low threshold for computed tomography scans in older patients presenting with a proximal femur fracture

**DOI:** 10.1007/s41999-024-01007-9

**Published:** 2024-06-19

**Authors:** S. van Westendorp, S. H. M. Robben, M. A. A. van Hooft, S. A. A. Dierckx, H. A. A. M. Maas

**Affiliations:** 1 Department of Geriatric Medicine, Elisabeth-Tweesteden Hospital, Tilburg, The Netherlands; 2 Emergency Department, Elisabeth-Tweesteden Hospital, Tilburg, The Netherlands

**Keywords:** Frailty, Proximal femur fracture, Low-energy trauma, CT scanning, Older people

## Abstract

**Purpose:**

Current guidelines recommend a low threshold for computerized tomography (CT) scanning in older patients presenting with low-energy trauma (LET). With the ageing of the population, this results in increased use of healthcare resources and costs. We aim to assess (1) the number of CT scans performed as part of the initial trauma screening, (2) their traumatic clinical implications, and (3) their non-traumatic clinical implications.

**Methods:**

A retrospective study in patients ≥ 70 years presenting at a Dutch trauma centre with a proximal femur fracture following a LET between 2021 and 2022. We collected data concerning demographics, Clinical Frailty Scale, Injury Severity Score, number of CT scans and whether the results of these scans altered clinical management.

**Results:**

We included 278 patients. Median age was 83.0 years (IQR 77.0–89.0), median ISS was 9 (IQR 9–10) and, most common mechanism of injury was a ground level fall (*n* = 159, 57.2%). In 49 patients (17.6%) one or more CT scans were performed. These scans did not reveal co-existing traumatic injuries altering clinical management. In 2 patients (0.7%) incidental findings were found that immediately affected treatment.

**Conclusion:**

Our study concludes that (1) approximately one in five patients with a proximal femur fracture received a CT scan as part of the initial trauma screening, resulting in (2) no traumatic and (3) minimal non-traumatic clinical implications. Therefore, a restrictive policy can be justified in patients with no additional clinical signs or symptoms and admission to the hospital. Further prospective research would be valuable to confirm our results.

**Supplementary Information:**

The online version contains supplementary material available at 10.1007/s41999-024-01007-9.

## Key summary points

**Aim:**

We evaluated the number of computerized tomography (CT) scans performed as well as the traumatic and non-traumatic clinical implications of using a low threshold for performing CT scans as part of the initial trauma screening in older patients presenting at the ED with a proximal femur fracture following a low-energy trauma (LET).

**Findings:**

Approximately one in five patients received a CT scan as part of the trauma screening in older patients with a proximal femur fracture after a LET. Results show no traumatic clinical implications, two non-traumatic clinical implications, and two initially missed injuries.

**Message:**

In this subgroup of older trauma patients admitted with a proximal femur fracture, a restrictive policy can be used instead of using a low threshold for CT scans as part of the initial trauma screening at the emergency department (ED).

**Supplementary Information:**

The online version contains supplementary material available at 10.1007/s41999-024-01007-9.

## Introduction

Falls are considered a major public health problem [1–5], with 32–42% of individuals aged ≥ 70 years experiencing at least one fall each year worldwide [3, 4]. Most falls in older patients are classified as low-energy trauma (LET). However, these falls often result in morbidity and mortality, as well as in the use of healthcare services and increasing healthcare costs [5–7]. Currently, 10–15% of all emergency department (ED) visits are related to a fall, and among older people, more than 50% need to be admitted because of a fall-related injury [5, 8, 9]. With the ageing of the population, it is expected that these numbers will continue to rise in the future [7].

Due to the increasing number of older patients presenting to the ED with a fall, several studies report that there is an increase in the number of computerized tomography (CT) scans performed following a LET [10–13]. However, international recommendations regarding the use of CT scans in older patients presenting at the ED after a LET vary. Recommended radiological assessments range from the routine performance of a total body CT scan to using a low threshold for performing CT scans [1, 14, 15]. The recommendation to use a low threshold for CT scanning is mainly based on expert opinion and consensus as high-quality literature is lacking [16]. Studies concerning the use of CT scans in older trauma patients are mainly performed in the US, where the healthcare system is different compared to most European healthcare systems. In addition, these studies focused solely on the utility of a specific type of CT scan, such as a CT head, and did not take into account the frailty of the studied population, or studied a heterogeneous population, which makes it difficult to generalize the results to daily practice [2, 13, 17–24].

Although CT scanning often results in valuable diagnostic information, it can also lead to incidental findings that need follow-up or additional diagnostic procedures, resulting in higher healthcare costs. Therefore, a critical attitude towards scanning remains necessary, partly due to the increasing scarcity in healthcare. Still, according to the Dutch guideline for initial radiographic screening in trauma patients, using a low threshold for CT scanning in older patients with a LET is recommended [16]. This is based on the assumption that a LET in older patients can result in remarkably severe injuries, due to changes in physiology, co-morbidity, and use of medication such as anticoagulants. Due to a decreased predictive value of signs and symptoms in older patients, triage can be challenging, leading to an underestimation of the severity of injuries. This can result in diagnostic and therapeutic delays. In addition, the high prevalence of degenerative disorders in the older population can make the interpretation of plain film radiography more difficult whereas the long-term effects of ionizing radiation are less of a concern [16]. However, except for recommending a low threshold for CT scanning in older trauma patients in general, the guideline does not provide more specific guidance for everyday practice. In addition, it is unknown whether this policy is justifiable for all older patients presenting with a LET.

In this study, we aim to assess the added value of using a low threshold for CT scanning in a specific group of trauma patients, i.e., older mostly frail patients admitted with a proximal femur fracture after a LET. First, we will assess the number of CT scans performed as part of the initial trauma screening. Second, we will investigate the traumatic clinical implications of using a low threshold for CT scanning by examining the impact traumatic findings on CT had on clinical management. Third, we will study the non-traumatic clinical implications by assessing incidental findings that immediately altered treatment.

## Methods

### Setting

This retrospective cohort study was performed in the Elisabeth-TweeSteden Hospital (ETZ), a level 1 trauma centre and teaching hospital in Tilburg, the Netherlands, between January 2021 and January 2022. As patient files were studied retrospectively, patients were included using the local opt-out procedure, i.e. data were available for scientific research unless a patient explicitly objected to use their electronic patient files for research purposes. The institutional review board of the ETZ, reviewed the study (study number: L1510.2022) and deemed it exempt from formal approval, as data collection followed routine clinical practice.

### Population

Patients were considered for inclusion if they were ≥ 70 years and admitted to the ETZ with a proximal femur fracture after a LET between January 2021 and January 2022. Furthermore, to have a complete data set, a geriatrician had to be involved during admission. Involvement of a geriatrician is consistent with the Dutch practice guideline for Comprehensive Geriatric Assessment, which states that a geriatrician should be involved in the care of patients ≥ 70 years admitted with a proximal femur fracture [25]. Following international standards, we defined a LET as having an injury severity score (ISS) of ≤ 15 [26]. The ISS is used for standardizing the severity of traumatic injuries. We used the clinical frailty scale (CFS) [27] to assess the level of frailty. Subsequently, we categorized individuals into two groups: (1) fit when the CFS was 1–4, and (2) frail when the CFS was ≥ 5 [28]. Patients with a proximal femur fracture due to a high-energy trauma as well as those with pathological fractures, periprosthetic fractures or distal femur fractures were excluded. Further, if patients presented themselves with a second femur fracture during the study period, we only included data from their first presentation.

### Data collection

Data were collected using the electronic patient files. We recorded information on patient characteristics, the use of anticoagulants, vital signs upon presentation, and the mechanism of injury. If the ISS or CFS were not explicitly mentioned, we retrospectively assigned scores based on the information documented in the patient files. Recent studies have shown that a retrospectively attained CFS is a valid instrument for measuring frailty [29, 30].

Following our research questions, we first collected the number of initial and secondary CT imaging, as well as initial plain film radiography. We recorded the results of this imaging and its indications.

Second, we assessed the traumatic clinical implications of CT imaging, which were defined as traumatic lesions other than the proximal femur fracture (co-existing traumatic lesions) on CT imaging that affected clinical management. To do this, we recorded the number and type of traumatic injuries diagnosed using CT imaging and assessed whether these findings affected clinical management. We created a list of possible co-existing traumatic lesions prior to the study (Supplementary Table S1). Additionally, we drafted a list of possible alterations in clinical management, categorized as minor, medium, or major alterations (Supplementary Table S2). These lists were based on the results of a study by Byrne et al. [31]. In this study, two reviewers defined management changes before the start of the study showing perfect interobserver agreement. These lists were adapted by the research team to better suit our research population, such as adding a category with medium alterations in clinical management to the list.

Third, we assessed the non-traumatic clinical implications of CT imaging, which were defined as previously unknown findings (incidental findings) other than any traumatic injuries caused by the LET, that immediately influenced treatment, such as an extensive malignancy or pneumonia. We recorded the number and types of incidental findings as well as how they immediately affected treatment.

Fourth, we recorded the number of missed traumatic injuries. Missed traumatic injuries were defined as traumatic injuries diagnosed after the completion of the assessment in the ED. The list of possible co-existing traumatic lesions (Supplementary Table S1) was used to identify missed traumatic injuries. If there was uncertainty regarding whether a finding on the CT scan was a co-existing traumatic lesion, the cause of the trauma, a complication of surgery, or an incidental finding, two members of the research team (SR, HM) independently reviewed these findings.

### Statistical analysis

Due to the descriptive nature of our study, we mainly used descriptive statistics. Patient characteristics were provided as medians with interquartile ranges (IQR) for continuous variables. For categorical variables, proportions and percentages were used. We used descriptive statistics such as numbers and percentages to report on outcomes concerning traumatic or incidental findings. In addition, we used a chi-square test to compare between groups when assessing the influence of frailty and anticoagulation on the use of CT imaging. Statistical analyses were performed using IBM SPSS Statistics, version 24 (IBM Corp., Armonk, N.Y., USA).

## Results

**Fig. 1:**
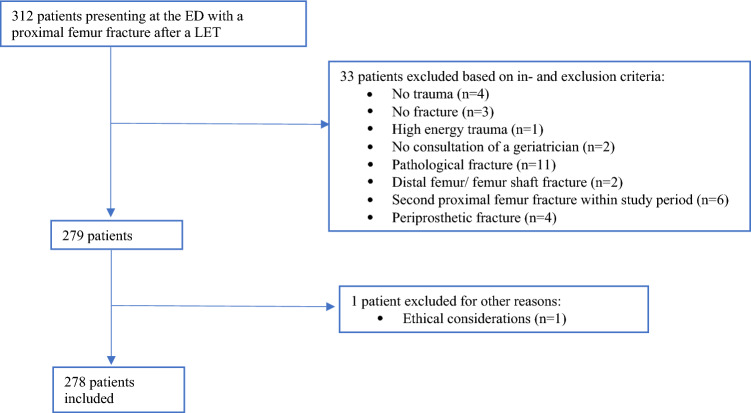
Flowchart of patient inclusion

We screened a total of 312 patients for inclusion; of these, 278 were included. Figure 1 shows a flowchart of the inclusion process.

The most common reason for exclusion was a highly suspected or proven pathologic fracture. One patient was excluded due to ethical considerations. An overview of patient characteristics is provided in Table 1. The median age of included patients was 83.0 years (IQR 77.0–89.0), 186 (66.9%) were female, and the majority of patients were considered frail, with a CFS ≥ 5 (*n* = 170, 61.2%). The median ISS was 9 (IQR 9–10), and the most common mechanism of injury was a ground-level fall (*n* = 159, 57.2%). 170 patients (61.2%) used anticoagulants, of which six (3.5%) used a combination. Most patients (*n* = 176, 63.3%) presented outside working hours (8:00–18:00).

**Table 1:** Patient characteristics

Variable	Participants (*n* = 278)
Age in years, median (IQR)	83.0 (77.0–89.0)
Female *n* (%)	186 (66.9)
Living situation before admission, *n* (%)	
Home without care	155 (55.8)
Home with care	50 (18.0)
Residential care	64 (23.0)
Other	9 (3.2)
Clinical Frailty Scale, *n* (%)	
1–4 fit	108 (38.8)
5–9 frail	170 (61.2)
One anticoagulant, *n* (%)	170 (61.2)
Vitamin K antagonist	35 (20.6)
DOAC	38 (22.4)
Acetylsalicylic acid	49 (28.8)
Clopidogrel	41 (24.1)
Other	1 (0.6)
Two anticoagulants, *n* (%)	6 (3.5)
Vitamin K antagonist + Clopidogrel	2 (1.2)
DOAC + Clopidogrel	1 (0.6)
Acetylsalicylic acid + Clopidogrel	1 (0.6)
Acetylsalicylic acid + Other	2 (1.2)
Type of proximal femur fracture, *n* (%)	
Femoral neck fracture	163 (58.6)
Pertrochantaric fracture	113 (40.6)
Subtrochantic fracture	2 (0.7)
Mechanism of injury, *n* (%)	
Ground-level fall	159 (57.2)
Fall from some height (≤ 3 m)	39 (14.0)
Syncope	16 (5.8)
Weakness	10 (3.6)
Neurological complaints	1 (0.4)
Unclear	49 (17.6)
Missing data	4 (1.4)
Presentation outside working hours*, *n* (%)	176 (63.3%)
MEWS at presentation**, median (IQR)	1 (1–2)
ISS***, median (IQR)	9 (9–10)
In-hospital mortality, *n* (%)	12 (4.3)

*Working hours are between 8 AM and 6 PM

**n = 277 due to missing data

***n = 176 due to missing data

*IQR* interquartile range, *DOAC* direct oral anticoagulants, *MEWS* modified early warning score, *ISS* injury severity score

49 (17.6%) patients received 88 CT scans as part of the initial trauma assessment. In nine patients, these CT scans followed an initial plain radiography or ultrasound, to further assess a potential traumatic lesion. In the group of 49 patients receiving a CT scan, patients were significantly more classified as frail (*n* = 37, 75.5%) compared to the 229 patients who did not receive a CT scan (*n* = 133, 58.1%); *p* = 0.023). In addition, the 39 patients receiving a CT head scan were significantly more likely to use anticoagulants (*n* = 31, 79.5%) compared to the 239 patients who did not receive a CT head scan (*n* = 139, 58.2%); *p* = 0.011). Most CT scans were CT head scans (*n* = 39), CT scans of the cervical spine (*n* = 32), and CT scans of the pelvis (*n* = 11). Table 2 provides an overview of all CT scans that were performed.

**Table 2:** Types and numbers of CT scans used in trauma screening

Type of CT scan	Number of initial CT scans (in *n* = 39 patients)	Number of CT scans after (suspected) traumatic lesion(s) on X-ray/ ultrasound (in *n* = 10 patients)
CT head	39	0
CT cervical spine	32	0
CT chest	1	2
CT abdomen	1	2
CT pelvis	0	11

**Fig. 2:**
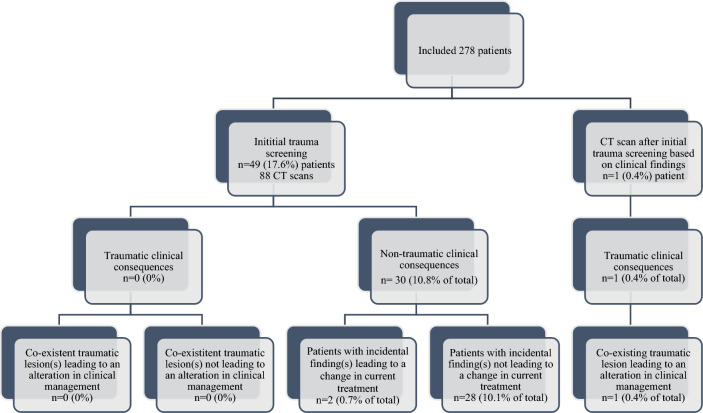
Traumatic and non-traumatic clinical implications of CT scans performed as part of the initial trauma screening and after initial trauma screening has been completed

Figure 2 provides a summary of the traumatic and non-traumatic clinical implications of the CT scans. There were no traumatic clinical implications, as zero co-existing traumatic injuries affected clinical management. In two (0.7%) patients incidental findings led to an immediate change in treatment. Both patients presented with clinical signs and symptoms warranting further evaluation. Further, 28 patients had incidental findings which did not immediately affect treatment. Findings included degenerative skeletal disorders, cortical brain atrophy, white matter disease, and signs of a previous stroke (see Table 3).

**Table 3:** Incidental findings and their clinical implications

Type of CT scan (n)	Incidental findings (*n*)*	Incidental findings that immediately affected treatment
CT head	Calcification (*n* = 1) Calcified meningioma (*n* = 1) White matter disease (*n* = 4) Previous cerebral infarction (n-7) Cortical atrophy (*n* = 12)	None
CT cervical spine	Pulmonary emphysema (*n* = 1) Previous fracture (*n* = 1) Calcified granuloma (*n* = 1) Degenerative disorders (*n* = 16)	None
CT chest—abdomen	Malignancy (*n* = 1) Tumour unknown malign or benign (*n* = 1) Possible pneumonia (*n* = 2) Decompensated heart failure (*n* = 1) Compression fracture (*n* = 1)	Extensive metastatic malignancy resulting in the start of end-of-life care (*n* = 1) Decompensated heart failure resulting in delay of surgery and consultation of a cardiologist (*n* = 1)

*Total *n* = higher than number of CT scans as one scan could result in multiple incidental findings

At presentation, eight patients had other traumatic injuries, such as a radius fracture, a humerus fracture or a rib fracture detected with plain film radiography. In two patients an additional traumatic injury was identified during admission based on clinical signs and symptoms. One of these patients developed nausea and vomiting after completion of the assessment in the ED. The CT head scan showed a subdural hematoma, resulting in the consultation of a neurologist and giving prothrombin complex concentrate to the patient. Based on our predefined list, these implications were defined as minor alterations in clinical management. The other patient complained of wrist pain after admission. Plain radiography showed a radius fracture.

## Discussion

This study evaluates the practice implications as well as the traumatic and non-traumatic clinical implications of using a low threshold for CT scanning as part of trauma screening in older patients with a proximal femur fracture after a LET. In a total of 49 patients (17.6%) one or more CT scans were performed. These scans did not reveal any co-existing traumatic injuries that altered clinical management. Additionally, in only two patients (0.7%) incidental findings immediately affected treatment. Last, in two patients an additional traumatic injury was identified during admission.

In other studies concerning the use of CT scanning in older patients presenting with a LET, the proportion of patients having a CT scan ranged from 7.2% [11] to 28% [10]. These studies differ from ours as they describe trends in CT utilization independent from using a low threshold for CT scanning and their study populations had no index-trauma as in the present study. Furthermore, these studies focused on specific types of CT scans, i.e. CT head scans instead of on all CT scans. Therefore, the present study provides more concrete data on a low threshold for performing CT scans in older patients after a LET. In our study, we found that 17.6% of older patients with a proximal femur fracture after a LET received a CT scan. As the recommendation to use a low threshold for CT scanning in older patients is rather general and does not state exactly which older patients should receive a CT scan, it is difficult to reflect on the implementation of this guideline. However, the number of CT scans made in this study and the fact that CT scanning in general was more often used in patients who are frail does suggest adherence to the guideline. This is supported by the fact that patients using anticoagulants received a CT head scan more often.

Three studies investigated the use of a panscan (head, neck, chest, and abdomen/pelvis) in different groups of trauma patients. The first retrospective study investigated the utility and implications of a panscan in older patients admitted to a trauma centre, following a ground-level fall. A panscan was associated with lesser hospital resource use but not with decreased mortality. Interestingly, more than 47% of patients in this study required admission to the Intensive Care Unit (ICU) and 56% had an ISS > 9 [20]. This suggests that those patients were more severely injured compared to the patients in our study. The second study focused on the use of a panscan for detecting intra-abdominal injuries in asymptomatic older patients suffering from a ground-level fall. They found that a panscan is not beneficial as it often detects injuries that are clinically insignificant, leading thus to increased use of hospital resources. The authors conclude that routine use of panscans in this population is a cause of unnecessary medical cost and radiation exposure and that selective CT scanning could reduce costs for hospitals [21]. In a third retrospective study, the authors compared the outcomes of a panscan versus selective CT scanning in older trauma patients. A sub-analysis categorized older patients with single injuries in three groups based on the most common mechanism of injury (falls < 2 m, falls > 2 and motor vehicle accidents). Across all groups, there was no significant difference in length of hospital stay, mortality and repeated CT scans for other suspected injuries between patients receiving a panscan and those who were only scanned selectively [24]. In conclusion, performing standard CT scans did in some studies result in increased use of hospital resources and costs, whereas it did not result in better outcomes in older patients with single injuries and a low ISS. This agrees with our findings as no CT scans in our study had any traumatic clinical implications in patients with a low ISS (mean 9) and a proximal femur fracture. However, there are notable differences between these and our study populations: as none of the three studies specifically included patients with a hip fracture, none of our patients were admitted to the ICU, and the mechanism of injury in our study was a fall from a maximum height of 3 m, which concurs with the definition of a LET by the American College of Surgeons [32].

Other studies evaluated the use of specific CT scans in older trauma patients. Sartin et al. studied the use of a CT head scan in hemodynamically stable patients ≥ 55 years with an initial Glasgow Coma Score of 15 presenting with a ground-level fall. The authors suggest that all these patients should have a CT head scan, as scanning resulted in a high proportion of treatment changes (21.7%), such as changes in medication (17.4%) and performing neurosurgery (4.3%). However, all patients with a positive finding on the CT scan complained of a headache or had a visible head injury [19]. Another study by Allen et al. specifically investigated the utility of CT head scans in geriatric patients with hip fractures following a LET. Out of 1864 patients, 502 (26.9%) received a CT head scan, which showed intracranial bleeding in 62 (12.3%). None of these patients required neurosurgical intervention, and only nine (14.5%) of patients with a positive CT head for acute intercranial bleed needed a modification in thromboprophylaxis following surgery. The authors concluded that CT head scans should be reserved for patients with a history or physical examination consistent with head trauma or who are using anticoagulation [22]. Next to brain injury, some studies indicate that older patients who fall from standing height are more prone to injuries of the upper cervical spine [18, 33]. A study by Heller et al. evaluated CT utilization including cervical CT scanning as part of completing the standard trauma screening in 101 minor trauma patients of all ages (mean age 59.7 years). Out of the 481 CT scans performed, seven (1.5%) found a clinically unsuspected injury, of which two were nondisplaced transverse process fractures. All the injuries were considered minor traumatic lesions with no clinical implications and no major traumatic issues in general [34]. This absence of clinical implications due to a traumatic lesion agrees with our results. In addition, in our study, no cervical spine lesions were found in even older patients with a minor trauma.

In our study, 30 (10.8%) out of the 278 patients had an incidental finding on their CT scan, which corresponds to incidental findings in 61.2% of the 49 patients who received an initial CT scan. Also, in 28 (93.3%) of the 30 patients, the incidental finding did not lead to an immediate alteration in clinical management. Other studies have reported a range of incidental findings [35–37]. For instance, a study by Niedermeier et al. found incidental findings in 73.9% of the patients receiving a CT scan as part of the emergency work-up of elderly patients with low-energy falls [36]. Incidental findings were most often discovered with a CT head, CT chest, and CT abdomen. However, these findings were considered harmless or asymptomatic in 83.6% of the patients.

Furthermore, incidental findings are more prevalent in whole-body CT scans [38]. A study investigating non-traumatic incidental findings on whole-body CT scans performed at the ED in patients with suspected multiple trauma found one or more incidental findings in 75,3% of the patients. Of these incidental findings 71.9% were considered most likely asymptomatic or harmless [35]. Even in a study by Lai et al. that discovered less incidental findings on CT scans performed at the ED in trauma patients (15.9%), most incidental findings were of minor concern (50.8%) or not urgent but in need of follow-up (47.2%) [37]. Therefore, although the number of incidental findings varied, the implications of these incidental findings was low across all studies. The variation in the number of reported incidental findings can be attributed to the use of different definitions of incidental findings between studies as well as by differences in the studied population and differences in the number of types of CT scans performed. In our study, the use of a CT chest and CT abdomen was limited and performing whole-body CT scans was not standard practice. This may explain the somewhat lower prevalence of incidental findings in our study compared to others.

Interpreting our results, we conclude that the traumatic and non-traumatic clinical implications of using a low threshold for CT scanning in patients with a proximal femur fracture following a LET are minimal. While we did not specifically focus on clinical signs and symptoms, the clinical implications of the CT scans performed as part of the trauma assessment were minimal, as in only two patients findings on the CT scans immediately altered clinical management. Both patients did have clear guiding signs and symptoms. Further, clinical symptoms resulted in additional diagnostics with relevant findings in two other patients during their hospital stay. This emphasizes the importance of clinical guiding signs and follow up. As all patients suffering a proximal femur fracture are admitted to the hospital, a controlled setting is provided for them where a wait-and-see policy is justifiable. In addition, as clinical follow-up is part of the usual care provided during a hospital admission limiting the number of CT scans in the ED could reduce healthcare costs and use of hospital resources, such as radiological equipment and staff. This can be especially valuable as most patients (*n* = 176, 63.3%) in our study presented at the ED outside of working hours when less staff is available. However, whether this strategy does indeed reduce costs and use of hospital resources should be evaluated in a prospective study. In addition, further confirmation of our results would be valuable in a prospective study, as it remains uncertain if the results of this study can be generalized to all older patients presenting at the ED with a LET without a reason for hospital admission.

This study has some limitations. First, it is retrospective in nature, as we cannot recover clinical reasoning or detailed information on performing CT scanning unless this was specifically described in the electronic patient files. Nevertheless, the clinical implications of performing CT scans were minimal. Second, data concerning the criteria for performing CT scans were scored by a single researcher, leading to a risk of bias. Third, because the study was a single-centre study, the number of patients involved was relatively small. Fourth, the period of inclusion coincided with the COVID pandemic, introducing a potential uncertainty about the implementation of using a low threshold. The policy of making a chest CT in patients with possible COVID may have resulted in a higher number of CT scans as well as a lower threshold for performing other types of CT scans. Last, as this study was conducted in a single country, the generalisability of this study primarily pertains to countries with similar healthcare infrastructure. In addition, the fact that a geriatrician was involved in the care of all patients in our study may have affected our results, which could have an impact on generalisability as well.

Nonetheless, this study also has several strengths. For one, this is the first study to examine the number of CT scans performed and the clinical implications of using a low threshold for CT scanning in older patients as part of everyday trauma assessment. As such, it provides insight into the number and types of CT scans performed in older patients presenting with a proximal femur fracture following a LET. Second, we focused on all types of performed CT scans rather than a specific CT scan. Third, the study was designed to reflect real-life conditions in a large peripheral level 1 trauma centre, making the results representative of daily practice. Fourth, the retrospective design provided us with the opportunity to have follow-up for injuries that were initially missed. Fifth, the lists of co-existing traumatic lesions and alterations in management were clearly determined beforehand, lowering the risk of abstractor bias. Last, our study highlights the importance of implementing care pathways for older patients with LET. This is of special importance in an era with an aging population and scarce healthcare resources.

## Conclusions

This study has shown that (1) in older patients presenting with a proximal femur fracture following a LET, a low threshold for CT scanning results in a CT scan in about one in five patients. However, (2) the traumatic and (3) the non-traumatic clinical implications of these CT scans were minimal. Therefore, we suggest reconsidering the current practice of using a low threshold for CT scanning in older, mostly frail patients presenting with a proximal femur fracture who are admitted to the hospital. In this population, a restrictive policy can be used for CT scanning, when follow-up on clinical signs and symptoms is provided. Such a policy may result in a reduction of healthcare costs and resources and may result in less time in the ED for frail older patients. However, it would be valuable to confirm our results in a prospective study, focusing on CT scanning, patient trajectories, and healthcare costs and use of resources. In addition, it would be worthwhile to broaden the scope of the study to include the complete population of frail older people presenting at the ED following a LET.

## Supplementary Information

Below is the link to the electronic supplementary material.

Supplementary file1 (DOCX 13 KB)

Supplementary file2 (DOCX 14 KB)

## References

[1] American College of Surgeons (ACS) (2013) Geriatric trauma management guidelines. https://www.facs.org/media/rddahzbb/geriatric_guidelines.pdf. Accessed 19 May 2023

[2] Khurrum M, Chehab M, Ditillo M et al (2021) Trends in geriatric ground-level falls: report from the national trauma data bank. J Surg Res 266:261–268. 10.1016/j.jss.2021.02.047 34034061 10.1016/j.jss.2021.02.047

[3] Salari N, Darvishi N, Ahmadipanah M, Shohaimi S, Mohammadi M (2022) Global prevalence of falls in the older adults: a comprehensive systematic review and meta-analysis. J Orthop Surg Res 17(1):334. 10.1186/s13018-022-03222-1 35765037 10.1186/s13018-022-03222-1 PMC9238111

[4] World Health Organisation (WHO) (2021) Falls https://www.who.int/news-room/fact-sheets/detail/falls. Accessed 19 May 2023

[5] World Health Organisation (WHO) (2007) Global report on falls prevention in older age. https://extranet.who.int/agefriendlyworld/wp-content/uploads/2014/06/WHo-Global-report-on-falls-prevention-in-older-age.pdf. Accessed 19 May 2023

[6] Hartholt KA, van Beeck EF, Polinder S et al (2011) Societal consequences of falls in the older population: injuries, healthcare costs, and long-term reduced quality of life. J Trauma 71(3):748–753. 10.1097/TA.0b013e3181f6f5e5 21045738 10.1097/TA.0b013e3181f6f5e5

[7] Estimation of the global prevalence of dementia in 2019 and forecasted prevalence in 2050: an analysis for the Global Burden of Disease Study 2019. Lancet Public Health 2022;7(2):e105-e125. 10.1016/s2468-2667(21)00249-8. 10.1016/S2468-2667(21)00249-8 PMC8810394 34998485

[8] Scuffham P, Chaplin S, Legood R (2003) Incidence and costs of unintentional falls in older people in the United Kingdom. J Epidemiol Community Health 57(9):740–744. 10.1136/jech.57.9.740 12933783 10.1136/jech.57.9.740 PMC1732578

[9] Do MT, Chang VC, Kuran N, Thompson W (2015) Fall-related injuries among Canadian seniors, 2005–2013: an analysis of the Canadian community health survey. Health Promot Chronic Dis Prev Can 35(7):99–108. 10.24095/hpcdp.35.7.01 26378768 10.24095/hpcdp.35.7.01 PMC4910457

[10] Brinjikji W, Kallmes DF, Cloft HJ (2015) Rising utilization of CT in adult fall patients. AJR Am J Roentgenol 204(3):558–562. 10.2214/ajr.14.13107 25714285 10.2214/ajr.14.13107

[11] Tong GE, Staudenmayer K, Lin F, Hsia RY (2016) Use of emergency department imaging in patients with minor trauma. J Surg Res 203(1):238–245. 10.1016/j.jss.2015.11.046 26732499 10.1016/j.jss.2015.11.046

[12] Bruls RJM, Kwee RM (2020) Workload for radiologists during on-call hours: dramatic increase in the past 15 years. Insights Imaging 11(1):121. 10.1186/s13244-020-00925-z 33226490 10.1186/s13244-020-00925-z PMC7683675

[13] Khaja A, Horný M, Balthazar P et al (2021) Disproportionate use in minor trauma is driving emergency department cervical spine imaging: an injury severity score-based analysis. J Am Coll Radiol 18(11):1532–1539. 10.1016/j.jacr.2021.07.006 34339664 10.1016/j.jacr.2021.07.006

[14] Atinga A, Shekkeris A, Fertleman M, Batrick N, Kashef E, Dick E (2018) Trauma in the elderly patient. Br J Radiol 91(1087):20170739. 10.1259/bjr.20170739 29509505 10.1259/bjr.20170739 PMC6221775

[15] Colwell C (2023) Geriatric trauma: initial evaluation and management. UpToDate. https://www.uptodate.com/contents/geriatric-trauma-initial-evaluation-and-management. Accessed 19 May 2023

[16] Federatie Medisch Specialisten (FMS) (2019) Initiële radiodiagnostiek bij traumapatiënten. https://richtlijnendatabase.nl/richtlijn/initi_le_radiodiagnostiek_bij_traumapati_nten/startpagina_-_initiele_radiodiagnostiek_bij_trauma_patienten.html. Accessed 19 May 2023

[17] Benayoun MD, Allen JW, Lovasik BP, Uriell ML, Spandorfer RM, Holder CA (2016) Utility of computed tomographic imaging of the cervical spine in trauma evaluation of ground-level fall. J Trauma Acute Care Surg 81(2):339–344. 10.1097/ta.0000000000001073 27454805 10.1097/ta.0000000000001073

[18] Lomoschitz FM, Blackmore CC, Mirza SK, Mann FA (2002) Cervical spine injuries in patients 65 years old and older: epidemiologic analysis regarding the effects of age and injury mechanism on distribution, type, and stability of injuries. AJR Am J Roentgenol 178(3):573–577. 10.2214/ajr.178.3.1780573 11856676 10.2214/ajr.178.3.1780573

[19] Sartin R, Kim C, Dissanaike S (2017) Discussion of: is routine head CT indicated in awake stable older patients after a ground level fall? Am J Surg 214(6):1055–1058. 10.1016/j.amjsurg.2017.07.038 28958650 10.1016/j.amjsurg.2017.07.038

[20] Dwyer CR, Scifres AM, Stahlfeld KR et al (2013) Radiographic assessment of ground-level falls in elderly patients: Is the “PAN-SCAN” overdoing it? Surgery 154(4):816–820. 10.1016/j.surg.2013.07.015 24074420 10.1016/j.surg.2013.07.015

[21] Gartin CG, Reyes J, Helmer SD, Haan JM (2019) Injury patterns and incidence of intra-abdominal injuries in elderly ground level fall patients: Is the PAN-SCAN warranted? Am J Surg 218(5):847–850. 10.1016/j.amjsurg.2018.11.041 30563694 10.1016/j.amjsurg.2018.11.041

[22] Allen J, Ravichandiran K, McLaughlin TL et al (2021) The utility of head CT scans in geriatric patients with hip fractures following a low energy injury mechanism: a retrospective review. Injury 52(6):1462–1466. 10.1016/j.injury.2020.12.022 33536129 10.1016/j.injury.2020.12.022

[23] Morrison J, Jeanmonod R (2014) Imaging in the NEXUS-negative patient: when we break the rule. Am J Emerg Med 32(1):67–70. 10.1016/j.ajem.2013.08.062 24094866 10.1016/j.ajem.2013.08.062

[24] Mohamed H, Teoh K (2021) Outcome of selective CT vs. pan-CT scan in elderly trauma patients: a retrospective cohort study in a level 1 trauma center. Chin J Traumatol. 24(5):249–254. 10.1016/j.cjtee.2021.04.010 33947622 10.1016/j.cjtee.2021.04.010 PMC8563841

[25] Federatie Medisch Specialsten (FMS) (2013) Comprehensive geriatric assessment (CGA). https://richtlijnendatabase.nl/richtlijn/comprehensive_geriatric_assessment_cga/startpagina_-_comprehensive_geriatric_assessment_cga.html. Accessed 19 May 2023

[26] Palmer C (2007) Major trauma and the injury severity score–where should we set the bar? Annu Proc Assoc Adv Automot Med 51:13–29 (**(In eng)**) 18184482 PMC3217501

[27] Rockwood K, Song X, MacKnight C et al (2005) A global clinical measure of fitness and frailty in elderly people. CMAJ 173(5):489–495. 10.1503/cmaj.050051 16129869 10.1503/cmaj.050051 PMC1188185

[28] Falk Erhag H, Guðnadóttir G, Alfredsson J et al (2023) The association between the clinical frailty scale and adverse health outcomes in older adults in acute clinical settings—a systematic review of the literature. Clin Interv Aging 18:249–261. 10.2147/cia.S388160 36843633 10.2147/cia.S388160 PMC9946013

[29] Stille K, Temmel N, Hepp J, Herget-Rosenthal S (2020) Validation of the clinical Frailty Scale for retrospective use in acute care. Eur Geriatr Med 11(6):1009–1015. 10.1007/s41999-020-00370-7 32770462 10.1007/s41999-020-00370-7

[30] Kay RS, Hughes M, Williamson TR, Hall AJ, Duckworth AD, Clement ND (2022) The clinical frailty scale can be used retrospectively to assess the frailty of patients with hip fracture: a validation study. Eur Geriatr Med 13(5):1101–1107. 10.1007/s41999-022-00686-6 35987870 10.1007/s41999-022-00686-6 PMC9553782

[31] Byrne R, Parks A, Hazelton JP, Kirchhoff M, Roberts BW (2020) Incidence and significance of injuries on secondary CT imaging after initial selective imaging in blunt trauma patients. Am J Emerg Med 38(8):1588–1593. 10.1016/j.ajem.2019.158432 31699428 10.1016/j.ajem.2019.158432

[32] Specialisten RFM. Licht traumatisch hoofd/hersenletsel (LTH). 2019 (https://richtlijnendatabase.nl/richtlijn/licht_traumatisch_hoofd_hersenletsel_lth/indicaties_ct_volwassenen_met_lth/minimaal_triviaal_hoofdletsel.html).

[33] Khanpara S, Ruiz-Pardo D, Spence SC, West OC, Riascos R (2020) Incidence of cervical spine fractures on CT: a study in a large level I trauma center. Emerg Radiol 27(1):1–8. 10.1007/s10140-019-01717-9 31463806 10.1007/s10140-019-01717-9

[34] Heller MT, Kanal E, Almusa O et al (2014) Utility of additional CT examinations driven by completion of a standard trauma imaging protocol in patients transferred for minor trauma. Emerg Radiol 21(4):341–347. 10.1007/s10140-014-1200-x 24532129 10.1007/s10140-014-1200-x

[35] Kroczek EK, Wieners G, Steffen I et al (2017) Non-traumatic incidental findings in patients undergoing whole-body computed tomography at initial emergency admission. Emerg Med J 34(10):643–646. 10.1136/emermed-2016-205722 28130347 10.1136/emermed-2016-205722

[36] Niedermeier S, Wania R, Lampart A et al (2022) Incidental CT findings in the elderly with low-energy falls: prevalence and implications. Diagnostics (Basel). 10.3390/diagnostics12020354 35204445 10.3390/diagnostics12020354 PMC8871195

[37] Lai WA, Liu PH, Tsai MJ, Huang YC (2020) Frequency, recognition, and potential risk factors of incidental findings on trauma computed tomography scans: a cross-sectional study at an urban level one trauma center. J Acute Med 10(3):106–114. 10.6705/j.jacme.202009_10(3).0002 33209569 10.6705/j.jacme.202009_10(3).0002 PMC7662100

[38] Treskes K, Bos SA, Beenen LFM et al (2017) High rates of clinically relevant incidental findings by total-body CT scanning in trauma patients; results of the REACT-2 trial. Eur Radiol 27(6):2451–2462. 10.1007/s00330-016-4598-6 27709280 10.1007/s00330-016-4598-6 PMC5408082

