# Supplementary material for: The clinical implications of using a low threshold for computed tomography scans in older patients presenting with a proximal femur fracture
Source: Eur Geriatr Med. 2024 Jun 19;15(4):1081–9. doi: 10.1007/s41999-024-01007-9 (PMC11377457; doi:10.1007/s41999-024-01007-9)
Supplement: Supplementary file 1 — Supplementary file1 (DOCX 13 KB) [file 41999_2024_1007_MOESM1_ESM.docx]

## Supplementary data

| **S1: List of co-existing traumatic lesions** |
| --- |
| Subdural hematoma |
| Epidural hematoma |
| Subarachnoid haemorrhage |
| Skull fractures |
| Intracerebral haemorrhage |
| Facial bone fractures |
| Spine fractures |
| Rib fractures |
| Pelvic fractures |
| Other fractures |
| Haemothorax |
| Pneumothorax |
| Pericardial fluid |
| Pulmonary embolism (causing the fall) |
| Pulmonary contusion |
| Pulmonary bleeding |
| Bleeding in an organ (i.e. liver, splenic, renal, other) |
| Other trauma-related injuries |
