# Supplementary material for: The clinical implications of using a low threshold for computed tomography scans in older patients presenting with a proximal femur fracture
Source: Eur Geriatr Med. 2024 Jun 19;15(4):1081–9. doi: 10.1007/s41999-024-01007-9 (PMC11377457; doi:10.1007/s41999-024-01007-9)
Supplement: Supplementary file 2 — Supplementary file2 (DOCX 14 KB) [file 41999_2024_1007_MOESM2_ESM.docx]

## Supplementary data

| **S2: List of minor, medium and major possible alterations in clinical management** | | |
| --- | --- | --- |
| *Minor* | *Medium* | *Major* |
| Adjustments in anticoagulation therapy (i.e. postponing anticoagulation/ thrombosis prophylaxis or discontinuation of anticoagulation) | Initiation of medication other than changes in anticoagulation therapy (e.g. antibiotics in case of an infection or corticosteroids in case of a subdural hematoma) | Blood product transfusion |
| Follow-up imaging with no major alterations in clinical management |  | Immobilisation for instance due to unstable spine fractures |
| Cast/orthopaedic appliance (e.g. neck brace/sling) |  | Postponing of hip surgery > 48 hours |
| Postponing of hip surgery < 48 hours |  | Cancellation of hip surgery |
| Consultation with another specialist |  | Transfer to ICU/ transfer to a specialty other than surgery/geriatrics |
|  |  | Neurosurgical Operation |
|  |  | Bedside procedure (e.g. chest tube insertion/ intubation) |
|  |  | Palliative care |
|  |  | Follow-up diagnostics other than scanning (e.g. puncture / endoscopy) |
|  |  | Embolisation |
